# Microsphere-Based Rapamycin Delivery, Systemic *Versus* Local Administration in a Rat Model of Renal Ischemia/Reperfusion Injury

**DOI:** 10.1007/s11095-015-1700-8

**Published:** 2015-05-09

**Authors:** Jurjen Zandstra, Marike M. van Beuge, Johan Zuidema, Arjen H. Petersen, Mark Staal, Luisa F. Duque, Sergio Rodriguez, Audrey A. R. Lathuile, Gert J. Veldhuis, Rob Steendam, Ruud A. Bank, Eliane R. Popa

**Affiliations:** Department of Pathology and Medical Biology, University of Groningen, University Medical Centre Groningen, Hanzeplein 1, 9713 GZ Groningen, The Netherlands; InnoCore Pharmaceuticals, Groningen, The Netherlands; Nanomi BV, Oldenzaal, The Netherlands

**Keywords:** ischemia reperfusion, kidney, local drug delivery, microspheres, rapamycin

## Abstract

**Purpose:**

The increasing prevalence and treatment costs of kidney diseases call for innovative therapeutic strategies that prevent disease progression at an early stage. We studied a novel method of subcapsular injection of monodisperse microspheres, to use as a local delivery system of drugs to the kidney.

**Methods:**

We generated placebo- and rapamycin monodisperse microspheres to investigate subcapsular delivery of drugs. Using a rat model of acute kidney injury, subcapsular injection of placebo and rapamycin monodisperse microspheres (monospheres) was compared to subcutaneous injection, mimicking systemic administration.

**Results:**

We did not find any adverse effects related to the delivery method. Irrespective of the injection site, a similar low dose of rapamycin was present in the circulation. However, only local intrarenal delivery of rapamycin from monospheres led to decreased macrophage infiltration and a significantly lower amount of myofibroblasts in the kidney, where systemic administration did not. Local delivery of rapamycin did cause a transient increase in the deposition of collagen I, but not of collagen III.

**Conclusions:**

We conclude that therapeutic effects can be increased when rapamycin is delivered subcapsularly by monospheres, which, combined with low systemic concentrations, may lead to an effective intrarenal delivery method.

**Electronic supplementary material:**

The online version of this article (doi:10.1007/s11095-015-1700-8) contains supplementary material, which is available to authorized users.

## Introduction

Acute kidney injury (AKI), caused for example by kidney transplantation or surgery-related ischemia/reperfusion, triggers inflammation irrespective of damage type. Inadequate treatment of the inflammatory phase after AKI leads to an excessive wound healing response, resulting in the deposition of large amounts of collagen (1,2). This fibrotic response occurring after insufficient recovery from AKI will lead to end stage renal disease and finally to kidney failure. In this final stage patients require renal replacement therapy, such as dialysis or kidney transplantation, which is costly and in the long term insufficient. For that reason, a solution should be sought in an early intervention during the inflammatory phase, in order to prevent fibrosis. To investigate whether this is possible we used an AKI rat model to see whether we can control the hostile microenvironment of the kidney. Although rats and humans differ in their response toward AKI we hypothesize that controlling the microenvironment after damage can lead to a better outcome.

Current treatments after AKI are based on slowing down or controlling the conditions that eventually may lead to chronic kidney disease, and include medication to decrease blood pressure and / or cholesterol, to reduce swelling or to treat anemia (3). After transplantation, oral medication needs to be taken in order to prevent rejection or inflammation (4). These treatments are not focused on treating the damage caused in the kidney by ischemic stress or other factors. This means that, even when the cause of kidney disease is being treated, the process of kidney disease may be ongoing.

Site-specific release of therapeutic compounds by means of an injectable drug delivery vehicle allows for optimal therapeutic effect at the site of interest, while minimizing systemic loss and undesirable side effects of the drug in the rest of the body, as reviewed by Kumar *et al.* (5). Biodegradable microspheres (MSP) are interesting vehicles for this type of drug delivery due to their capacity to release drugs controllably with respect to duration and dosage (6). Previously we have examined the use of differently sized MSP, and hypothesized that monodisperse MSP (mMSP) of 30 μm diameter would be best suited for drug delivery (7). This technique also seems promising in the treatment of AKI, since mMSP can be injected subcapsularly, leading to local release directly into the kidney. We propose to deliver existing drugs against kidney disease, since these drugs are often difficult to dose, requiring frequent blood monitoring and dose adjustments (8) to reduce or prevent adverse effects. A more gradual release of drug released locally from mMSP may prevent high blood levels and thus prevent side-effects.

Rapamycin has been shown to reduce inflammation in kidney injury models (9,10). Based on these findings we now hypothesized that subcapsular delivery of rapamycin, using mMSP can modulate the renal microenvironment in an AKI model (ischemia/reperfusion injury, IRI) in rats.

## Materials and Methods

### Preparation of Placebo and Rapamycin-Loaded Monospheres

MSP were prepared using SynBiosys 20[PDLA-PEG1000]-80[PLLA], a multiblock copolymer consisting of 20% *w/w* of poly(DL-lactide)-PEG1000-poly(DL-lactide) with a molecular weight of 2000 g/mol and 80% *w/w* of poly(L-lactide) with a molecular weight of 4000 g/mol (InnoCore Pharmaceuticals, Groningen, The Netherlands).

Monodisperse MSP (monospheres, mMSP) were prepared by a membrane emulsification-based solvent extraction/evaporation process using an Iris-20 microsieve membrane with uniformly sized pores of 20 μm (Nanomi BV, Oldenzaal, The Netherlands).

For placebo (drug-free) mMSP approximately 3.0 g of 20[PDLA-PEG1500]-80[PLLA] polymer was dissolved in 9 mL dichloromethane (DCM, p.a. stabilized with EtOH, Across, Geel, Belgium) as to obtain a 20% *w/w* solution and filtered through a 0.2 mm PTFE filter. The filtered polymer solution was processed through the microsieve membrane using 35 mbar air-pressure into an aqueous solution containing 4% *w/v* polyvinylalcohol (PVA 13–23, Sigma-Aldrich, Zwijndrecht, the Netherlands) as emulsifier thereby forming a dispersion of mMSP. This dispersion was stirred for at least 3 h at room temperature to extract and evaporate the solvent. The hardened MSP were concentrated by filtration and washed repeatedly with ultrapure water containing 0.05% Tween20 (Across) and finally lyophilized.

For rapamycin-loaded MSP, rapamycin (Sirolimus, LC Laboratories, Woburn, USA) was co-dissolved with the 20[PDLA-PEG1500]-80[PLLA] polymer to obtain a solution containing 20% *w/w* 20[PDLA-PEG1500]-80[PLLA] and 5% *w/w* rapamycin, which was used to prepare MSP using the same procedures as described above. Placebo mMSP and rapamycin-loaded mMSP were stored at −20°C until evaluation.

### Rapamycin Content of Monospheres

Rapamycin loading was determined by immersing mMSP (5–10 mg) in 600 μL of acetone:ethanol (2:1 *v/v*) to extract rapamycin. After 1 h, the mMSP were centrifuged at 10,000*g* for 20 min, whereafter the rapamycin concentration of the supernatant was determined by HPLC. HPLC was performed on a Waters 2695 Alliance system (Etten-Leur, The Netherlands) consisting of a 2998 Photodiode array detector and a computer with Empower 2 Software. Prior to analysis, the samples were filtered over an 0.2 μm PTFE filter. Separation was perfomed using an Xterra RP18 (4.6 × 150 mm, 3.5 μm) reverse phase column (Waters, Etten-Leur, The Netherlands) at 50°C using a flow rate of 1.0 mL/min and an injection volume of 20 μL. The mobile phase was acetonitrile:water (70:30 *v/v*). Rapamycin was detected by UV absorption at a wavelength of 278 nm. The standard curve of rapamycin was established and the concentration of unknown samples was calculated from the standard curve. The linearity was 0.999 in the range of 0.5 μg/g to 500 μg/g. Rapamycin MSP contained 18.4% *w/w* of rapamycin, representing an encapsulation efficiency of 99.1%.

### Animals and mMSP Administration Procedures

Male, 9 to 11 weeks old F344 rats (Harlan Laboratories, Inc. Livermore, USA), weighing 240 ± 50 g, underwent unilateral left kidney ischemia reperfusion injury (IRI) by clamping the renal artery and vein for 45 min. Prior to clamping, two subcapsular pockets were generated on the left kidney, in which either placebo mMSP, low-dose rapamycin-mMSP (0.37 mg rapamycin, obtained by mixing rapamycin MSP with placebo MSP 1:5), or high-dose rapamycin-mMSP (1.84 mg rapamycin) were injected (5 mg MSP/pocket). Alternatively, placebo-MSP or high-dose rapamycin-MSP were injected subcutaneously on the back of rats with unilateral IRI, to achieve systemic drug delivery. All rats were sacrificed after 7 or 14 days (*N* = 7/group for subcapsular implantations and *N* = 5/group for subcutaneous implantations). Kidneys were flushed with saline and fixed in Zinc fixative (0.1 M Tris-buffer, 3.2 mM calcium acetate, 23 mM Zinc acetate, 37 mM zinc chloride, pH 6.5–7.0; Merck) overnight, prior to paraffin embedding. Kidney samples were cut into 4.0 μm-thick sections. Blood samples were collected from each rat on day 0, 3, 7, 10 and 14 by orbital puncture and collected in EDTA tubes. Rapamycin amounts in the blood were determined by LC-MS/MS mass spectrometry using a previously described method (11) and [13C,2H3]-Sirolimus (SAS Alsachim, Illkirch, France) as an internal standard performed by the Mass Spectometry Core Facility (UMCG Groningen, The Netherlands). Plasma creatinine levels were determined according to standard procedures (Clinical laboratory UMCG Groningen, The Netherlands).

### (Immuno)histochemistry

Paraffin sections were dewaxed in xylene and rehydrated in ethanol. Characteristics of the primary antibodies used are summarized in Table I. Heat-induced antigen retrieval was performed using a 0.1 M Tris–HCl buffer, pH 9 (α-SMA, ED1) or 10 mM citrate buffer, pH 6.0 (LC-3). Enzymatic antigen retrieval was performed using protease K (collagens type I and III). Washing and blocking of aspecific binding sites, endogenous peroxidases and endogenous biotin was performed according to standard procedures. Stainings for ED1, α-SMA and LC-3 were visualized using 3-Amino-9-ethylcarbazole (AEC; Sigma-Aldrich, Zwijndrecht, Netherlands), stainings for collagens I and III were visualized using a Vector Red kit according to the manufacturer’s instructions (Vector Laboratories, Burlingame, CA). All tissue sections were counterstained with hematoxylin (Merck, Darmstadt, Germany) and mounted in Kaiser’s glycerin-gelatin (Merck).

**Table I:** Antibodies Used in this Study

	Manufacturer	Dilution
Primary antibody		
Mouse-anti-rat ED1	AbD Serotec	10 μg/ml
Mouse anti-α-SMA	Clone 1A4, DAKO	0.44 μg/ml
Mouse anti-collagen I	Abcam	1 μg/ml
Mouse anti-collagen III	Abcam	0.64 μg/ml
Rabbit anti-LC3	Novus Biologicals	20 μg/ml
Secondary antibody		
Rabbit-anti-mouse-HRP	DAKO	13 μg/ml
Rabbit-anti-mouse HRP	DAKO	13 μg/ml
Goat-anti-mouse-biotin	DAKO	5 μg/ml
Goat-anti-mouse-biotin	DAKO	5 μg/ml
Goat-anti-rabbit-biotin	DAKO	8.2 μg/ml

General histological assessment of the tissue reaction towards the mMSP was based on a periodic acid-Schiff staining, according to a standard staining protocol (Department of Pathology, University Medical Centre Groningen, Groningen, The Netherlands). Tissue sections were mounted in permount (Fisher Scientific International).

### Quantification of Stainings

Stainings were evaluated using a Leica DM 2000 microscope. For morphometric quantifications, five representative photomicrographs at 20× magnification were taken per section, using a Multispectral Imaging Camera (Perkin Elmer, Cambridge, UK). All photomicrographs were taken directly underneath the site of implantation of the microspheres, or at an equivalent site in the kidneys of rats that had received a subcutaneous implant. The photomicrographs were analysed using Nuance 3.0 software (Perkin Elmer). Stained areas were quantified and expressed as average surface area in square micrometer per high power field (μm2/HPF).

### Statistics

Statistical differences between groups were determined using a Kruskal–Wallis test, followed by a Dunn’s Multiple Comparison test to examine differences between each placebo and rapamycin combination separately. GraphPad Prism version 5 (GraphPad Software, La Jolla, USA) was used for all calculations. P values < 0.05 were considered to be statistically significant. Data are shown as mean ± standard error of the mean (SEM).

## Results

### Rapamycin Release

**Fig. 1:**
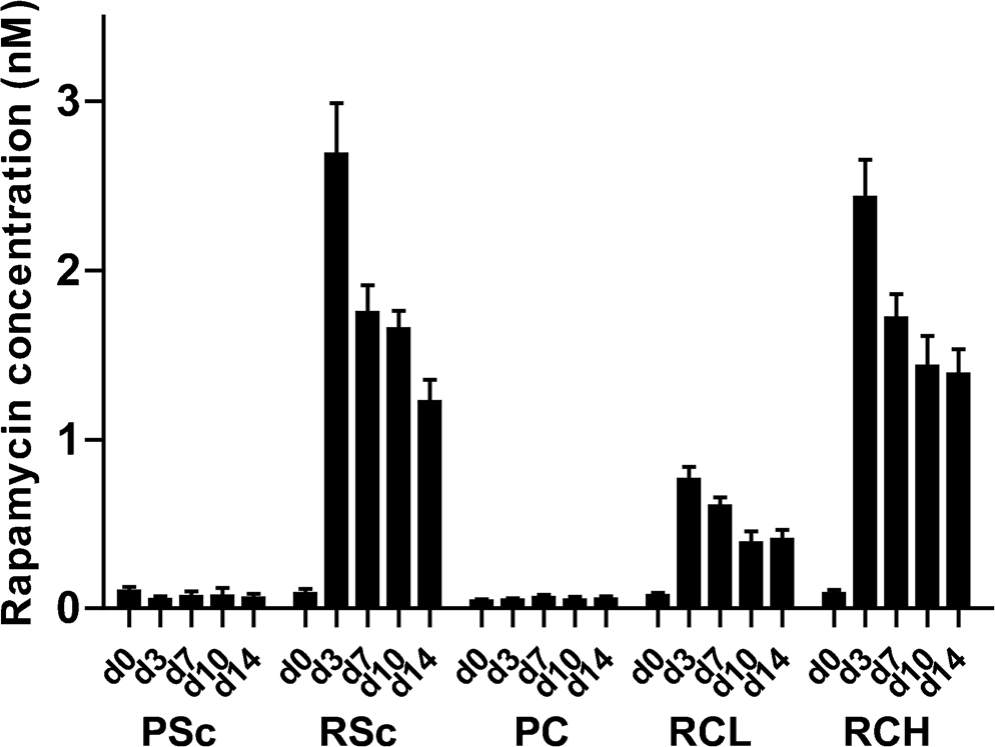
Blood levels of rapamycin. Blood rapamycin concentrations were determined in whole blood by HPLC mass spectrometry. *Bars* represent mean values and SEM. *Scale bar* represent 100 μm. *Abbreviations*: *PSc* placebo subcutaneous, *RSc* rapamycin subcutaneous high dose, *PC* placebo subcapsular, *RCL* rapamycin low dose, *RCH* rapamycin subcapsular high dose.

The measurement of rapamycin concentrations in the circulation provides insight into the *in vivo* release of rapamycin from the mMSP. Release of rapamycin into the circulation was detected in all three rat groups treated with rapamycin-containing mMSP and showed a dose dependency, as seen when comparing the subcapsular low dose (RCL) with the high dose (RCH). The highest blood levels of rapamycin were 2.5–3 nM, which were observed on day 3, and decreased subsequently in time. Release of rapamycin was sustained for at least 14 days. Interestingly, in rats implanted with the high dose rapamycin mMSP either subcutaneously or subcapsularly, there was no difference in blood rapamycin levels (Fig. 1). Since our effect studies did not show significant differences between RCL and RCH groups, or control and RCL groups (Suppl. Fig. 1), the remainder of the paper will focus on the effects of subcapsular and subcutaneous delivery of high dose rapamycin, hereafter defined as local and systemic delivery respectively.

### Effect of Subcapsular Injection of mMSP on Kidney Morphology and Function

**Fig. 2:**
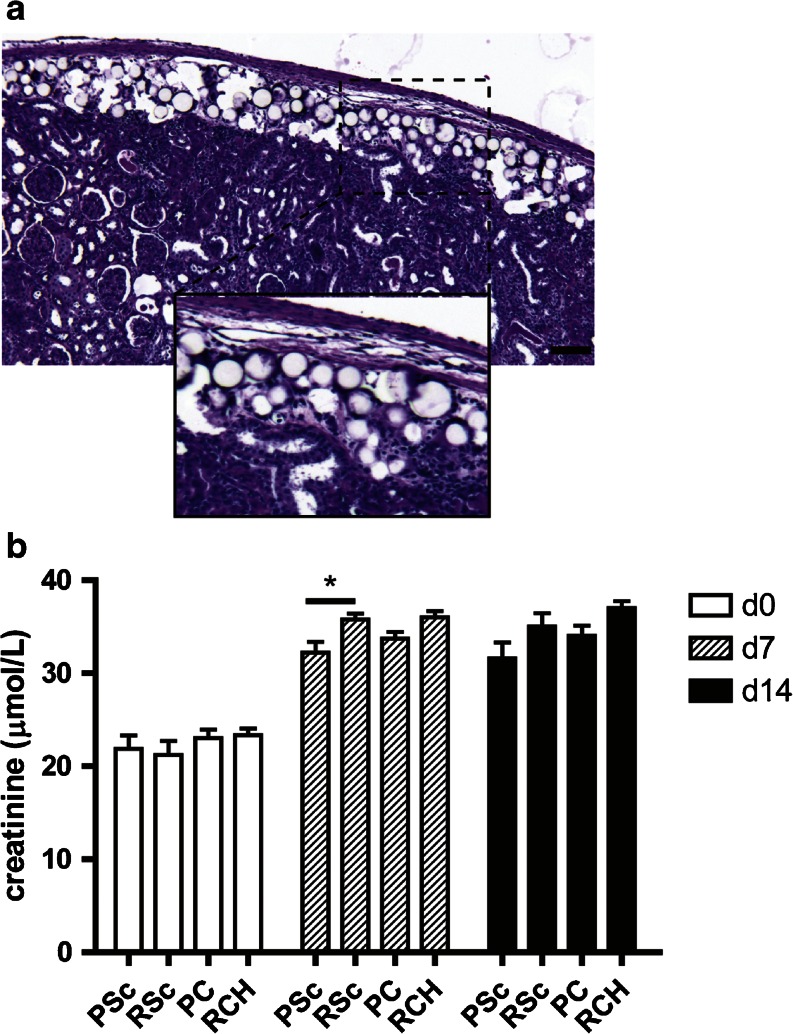
Effect of subcapsular injection of (Rapamycin) mMSP on kidney function. Placebo mMSP and rapamycin mMSP were injected subcapsularly in a model of IRI and explanted on day 7. Placebo mMSP are visible as *white spheres* localized between kidney capsule and kidney cortex (**a**). Kidney function was determined based on plasma creatinin levels (**b**). *Abbreviations*: *PSc* placebo subcutaneous, *RSc* rapamycin subcutaneous high dose, *PC* placebo subcapsular, *RCH* rapamycin subcapsular high dose.

To determine the effects of injecting mMSP underneath the kidney capsule on kidney morphology we performed a PAS staining (Fig. 2a). The PAS staining showed a slightly thickened kidney capsule, which may be caused by the technique used to create subcapsular pockets. However, more importantly, the injection of mMSP itself did not alter kidney cortex morphology in any way, compared to rats that had also undergone ischemia-reperfusion but were not injected with mMSP subcapsularly.

In addition, mMSP subcapsular injections did not have a negative effect on kidney function, as revealed by plasma creatinine levels (Fig. 2b). These findings are very important because if local drug delivery in the kidney is to be a feasible future treatment, the injection method itself should not disturb kidney function or morphology.

### Macrophage Infiltration and Presence/Formation of Myofibroblasts

**Fig. 3:**
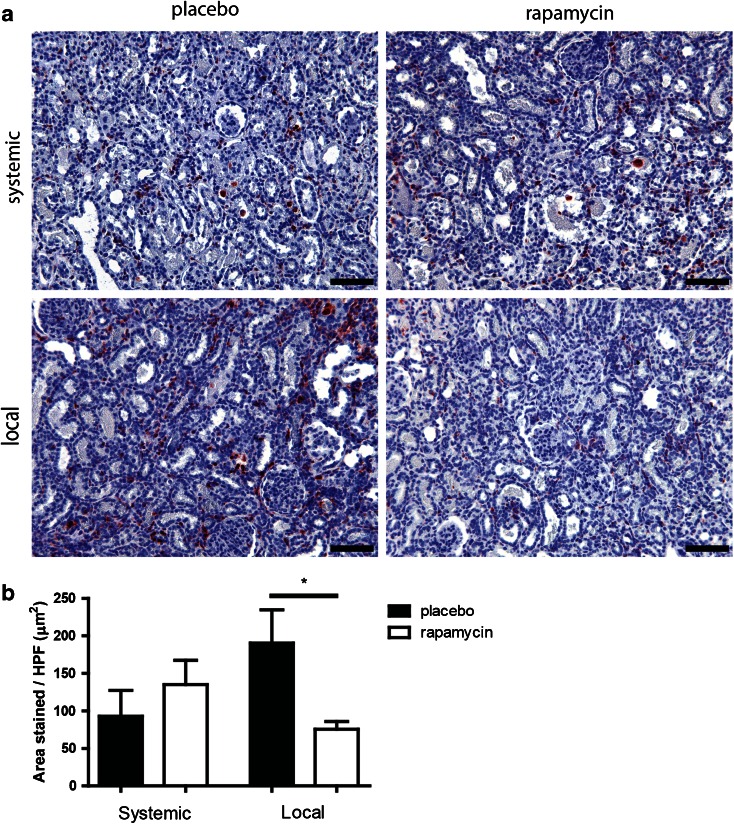
Renal interstitial macrophage infiltration after subcutaneous and subcapsular delivery of rapamycin or placebo. Placebo mMSP and rapamycin containing mMSP were injected subcutaneously and subcapsularly in a model of IRI and explanted at day 7. ED-1 staining was performed to assess renal interstitial macrophage infiltration (**a**). Average macrophage area was determined by morphometry (**b**). *Scale bars* represent 100 μm. *Bars* represents mean values and SEM. * *P* < 0.05, *HPF* high power field.

The inflammatory response after ischemia reperfusion injury (IRI) is predominantly an innate immune response which is characterized by polymorphonuclear (PMN) cell infiltration in the early phase, followed by macrophage infiltration later in IRI (12). We focused on macrophage infiltration, a key marker of the later inflammatory response. Local release of rapamycin by mMSP led to a lower macrophage influx compared to placebo mMSP (Fig. 3a), measured as ED-1 staining. No decrease of ED-1 was observed after systemic delivery of rapamycin *via* subcutaneous administration of rapamycin mMSP. The same trend was observed on day 14 (Suppl. Fig. 2A), when local delivery of rapamycin also decreased macrophage influx while systemic delivery did not.

### Myofibroblast Accumulation

**Fig. 4:**
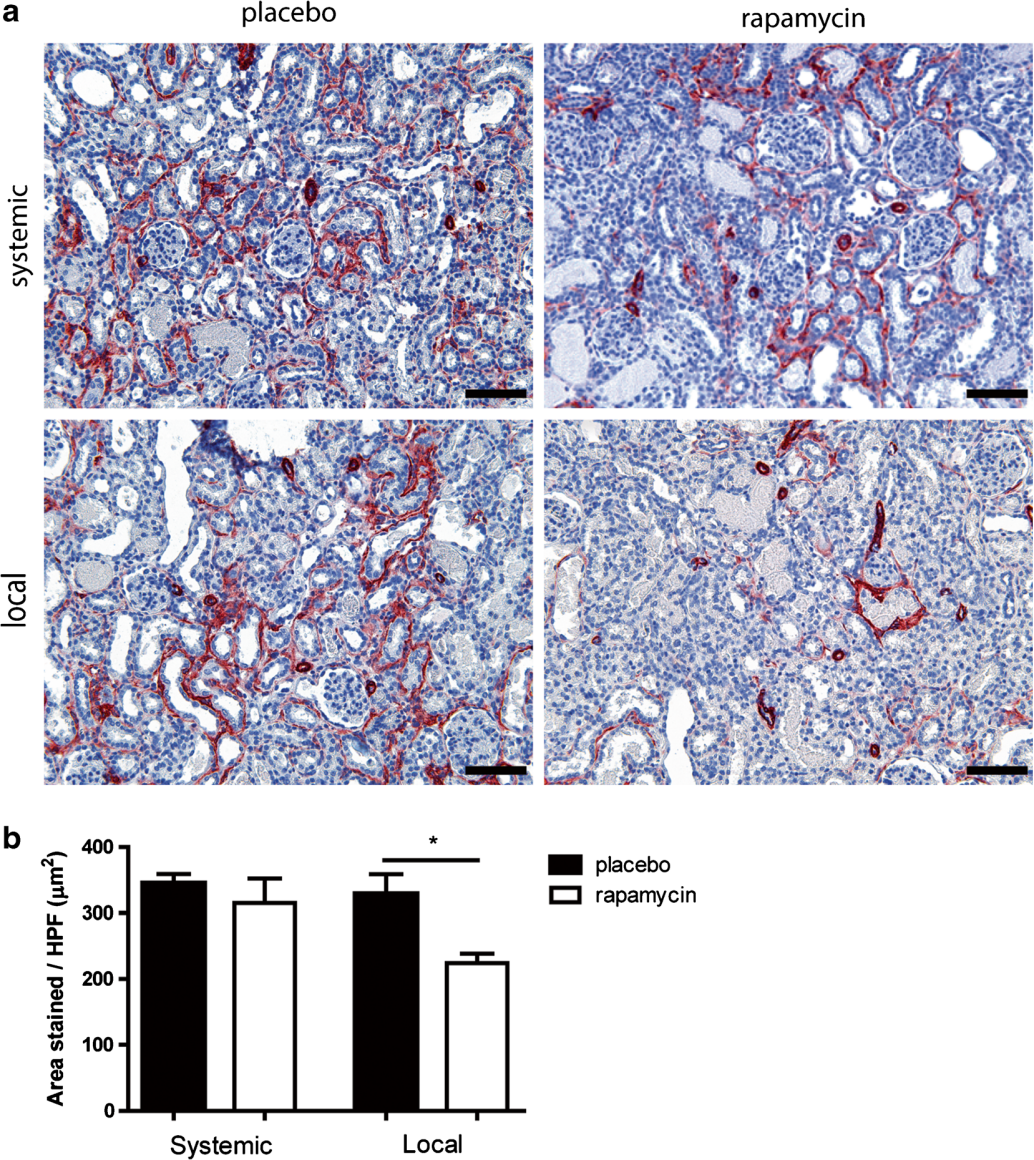
Renal interstitial myofibroblast accumulation after subcutaneous and subcapsular delivery of rapamycin or placebo. Placebo MSP and rapamycin MSP were injected subcutaneously and subcapsularly in a model of IRI and explanted at day 7. αSMA staining was performed to detect renal interstitial myofibroblasts (**a**). Average myofibroblast area was determined by morphometry (**b**). *Scale bars* represent 100 μm. *Bars* represents mean values and SEM. *HPF* high power field.

Upon damage, fibroblasts can be activated into myofibroblasts, which deposit collagens as a way of strengthening the damaged tissue. In normal wound-healing these cells undergo apoptosis after healing is complete. Although rats recover quickly from IRI damage, without long term collagen accumulation, the presence of myofibroblasts in the short term is still an important marker for tissue damage. Therefore we performed an immunostaining (Fig. 4a) for myofibroblasts (α-SMA) and found a significant decrease in α-SMA positivity when rapamycin was administered locally, but not when it was administered systemically (Fig. 4b). This significant difference was no longer present on day 14 (Suppl. Fig. 2B).

### Proliferation

It is known that rapamycin has anti-proliferative effects (13), therefore we examined whether a decrease in proliferation could explain the decrease seen in both ED-1 and α-SMA expression (see above) after local rapamycin treatment. We found no differences in Ki-67 positive cells between placebo and rapamycin treated rats (data not shown).

### Collagen Deposition

**Fig. 5:**
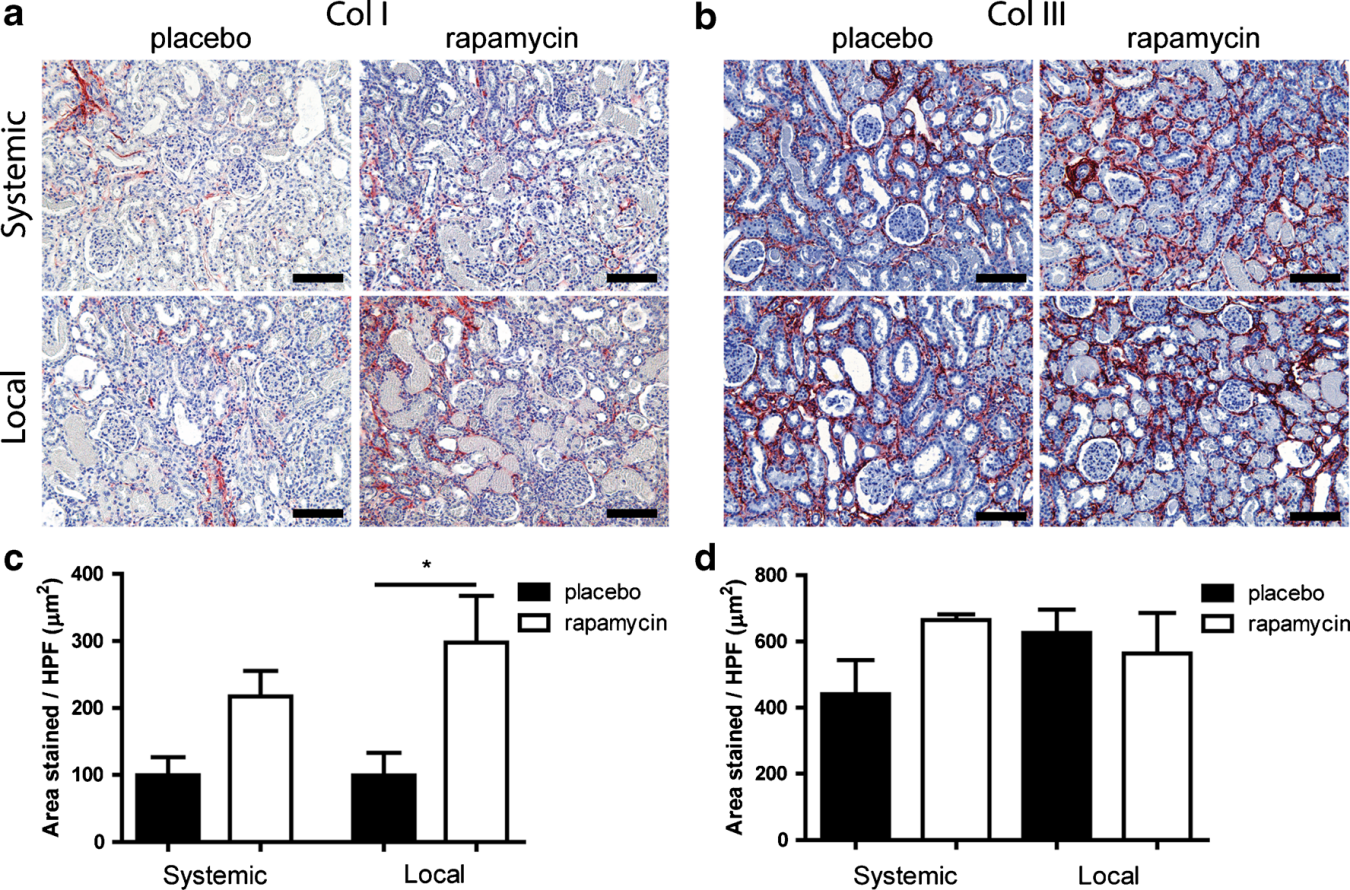
Renal deposition of collagen I and III after subcutaneous and subcapsular delivery of rapamycin or placebo. Placebo MSP and rapamycin MSP were injected subcutaneously and subcapsularly in a model of IRI and explanted at day 7. Collagen I (**a**), III (**b**) stainings were performed in order to assess renal interstitial extracellular matrix deposition. Average Collagen I and III area were determined by morphometry (**c** and **d** respectively). *Scale bars* represent 100 μm. *Bars* represents mean values and SEM. * *P* < 0.05, *HPF* high power field.

In response to ischemic injury, extracellular matrix is deposited in the kidney interstitium by myofibroblasts. We characterized the effects of local and systemic treatment with rapamycin on the deposition of two main types of collagen, *i.e.,* collagen I and III, by immunostaining. Deposition of collagen type I on day 7 was significantly higher after local treatment with rapamycin compared to placebo-MSP, while no significant effect of systemic rapamycin treatment was seen (Fig. 5a). On day 14 the difference between placebo and rapamycin-loaded MSP local treatment was still visible (Suppl. Fig. 2C). No significant differences were found in the deposition of collagen type III at any time point after local or systemic delivery (Fig. 5b and Suppl. Fig. 2D).

### Autophagy

**Fig. 6:**
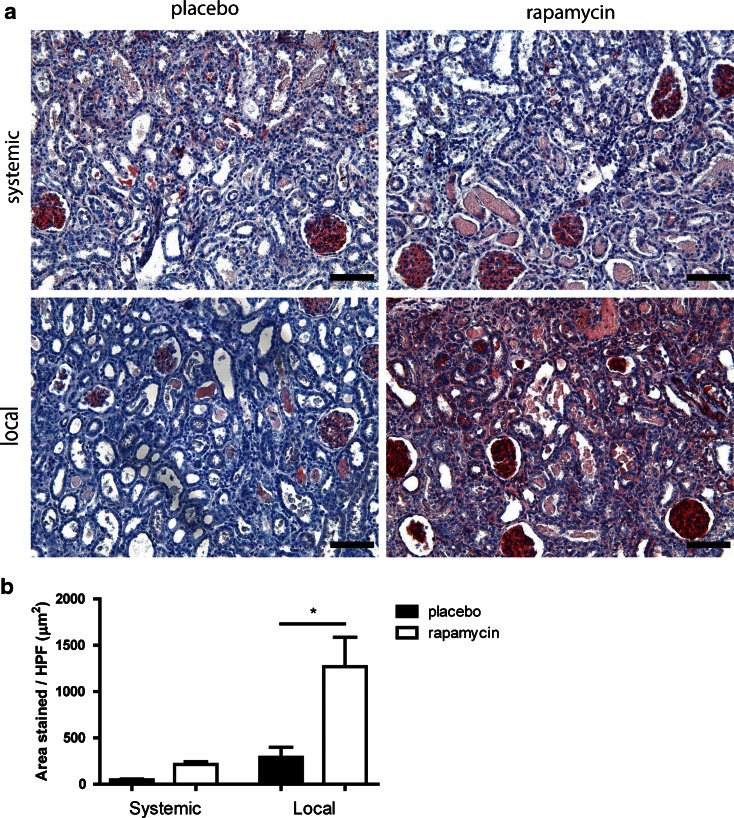
Renal expression of autophagy marker LC3 after subcutaneous and subcapsular delivery of rapamycin or placebo. Placebo MSP and rapamycin containing MSP were injected subcutaneously and subcapsularly in a model of IRI and explanted at day 7. LC3 staining was performed in order to detect autophagy induction as a marker for rapamycin release (**a**). Average LC3 area was determined by morphometry (**b**). *Scale bars* represent 100 μm. *Bars* represents mean values and SEM. * *P* < 0.05 *HPF* high power field.

Rapamycin has been described to directly induce autophagy, which has also been reported to be a factor in the recovery after AKI. We assessed protein levels of LC-3, a component of the autophagosome, in kidney sections of rats treated systemically and locally with rapamycin (Fig. 6a). Levels of LC-3 were slightly increased after systemic delivery of rapamycin. However, this increase was only significant after local rapamycin delivery (Fig. 6b), indicating an increased rapamycin-related drug effect in kidneys treated locally with rapamycin on day 7. On day 14 no significant differences were present anymore (Suppl. Fig. 2E).

## Discussion

This study shows that rapamycin can be delivered subcapsularly by means of sustained release mMSP over a period of 2 weeks. The mMSP successfully released rapamycin in a time-dependent manner into the circulation and into the kidney. Both the subcutaneous as well as the subcapsular administration route resulted in very low systemic levels of rapamycin, with similar *in vivo* release patterns, suggesting that drug release from these mMSP is not altered by the injection site. Furthermore, the effects seen in the kidney were not due to prolonged exposure to low systemic drug levels, but specifically caused by the local depot, since the subcutaneous delivery route did not result in major alterations in kidney recovery after IRI. Injection at either site led to systemic rapamycin concentrations below therapeutic levels, which are approximately 5–8 ng/ml in humans (8) and approximately 10 ng/ml in rats (14). Using identical doses of rapamycin, effects in the kidney were significantly more pronounced when the drug was delivered subcapsularly, than when it was delivered subcutaneously.

Notably, we still found detectable levels of rapamycin in blood 14 days after implantation of mMSP, although previous *in vitro* studies using similar mMSP indicated that all rapamycin would be released within 7 days (15). Since the reported half-life of rapamycin in rats is approximately 31 h (16), the sustained blood levels are unlikely to be caused by circulating rapamycin. Therefore, other factors related to the release of rapamycin *in vivo* may be responsible for the presence of rapamycin up to at least 14 days after implantation of the mMSP. Falke *et al.* (15) also reported *in vivo* plasma levels of rapamycin 7 days after subcapsular implantation of mMSP to be below 2 μg/L. At this time-point, using a different method, we found a rapamycin concentration in blood of 1.5–2 nM (corresponding to 1.4–1.8 μg/L) which shows the levels in our study to be in accordance with previously published results.

A recent study also showed beneficial effects of subcapsular delivery of rapamycin in a unilateral ureteral obstruction model (15), which causes renal fibrosis. This study also found a significant decrease in side-effects after subcapsular administration, since they found a loss of bodyweight only in their control group, where we found no systemic side effects connected to either the rapamycin subcutaneous or subcapsular treatment. However, in the previous study subcapsular delivery was compared to daily i.p. injections of rapamycin, whereas our study used subcutaneous depots as a control, ensuring comparison of continuous delivery and avoiding peaks in blood rapamycin levels.

The method itself, consisting of the injection of mMSP underneath the kidney capsule (between capsule and cortex) has been refined compared to current subcapsular injections (17) and did not induce cortical damage at the site of implantation, or affect kidney function. Notably, in contrast to this earlier study, we performed implantation of mMSP in kidneys which also underwent IRI, showing that the procedure is safe in damaged kidneys as well.

Our results show that local subcapsular drug delivery of rapamycin in a model of IRI results in reduced macrophage and myofibroblast numbers. The question arises whether this decrease is due to reduced infiltration or reduced proliferation of macrophages. Although rapamycin has anti-proliferative effects (13), Ki-67 staining within our experimental groups revealed no differences in cell proliferation in the kidney between any of the experimental groups. This suggests that local rapamycin delivery directly decreases macrophage influx or recruitment into the kidney, rather than inhibiting local proliferation. The decrease in myofibroblasts may be a secondary effect to the decrease in macrophages. It has been demonstrated that macrophages can produce highly fibrogenic growth factors such as transforming growth factor-β and platelet derived growth factor (18). A decrease in the amount of macrophages could thus lead to a decrease in the cytokines that induce activation of fibroblasts into myofibroblasts.

We hypothesized that an early intervention in the acute phase of the kidney injury would provide us with a valuable tool to inhibit the progression towards kidney fibrosis. In this study we succeeded in decreasing the inflammatory response, and we successfully reduced the amount of myofibroblasts in the kidney, which are considered to be the main collagen producing cells (19–21). However, this therapeutic effect did not result in decreased interstitial collagen deposition, but in an increase in collagen type I. A previous study reported that rapamycin may delay recovery after IRI (22), showing that normalization of GFR and proliferation of tubular epithelium occurred at a later time-point, but this study did not address collagen deposition. Possibly in the present study the delay in recovery is reflected in higher collagen levels in rapamycin-treated kidneys compared to those treated with placebo. In this respect, it may be notable that all rapamycin-treated groups showed a slight increase in blood creatinin levels, although this increase was only significant in rats implanted with mMSP subcutaneously, which did not show a significant increase in collagen. Macrophage and myofibroblast presence were determined based on expression of ED-1 and α-SMA respectively. These cell-type markers are conceivably more transient than collagen, once it is deposited. The discrepancy between high collagen levels and decreased α-SMA positive myofibroblasts may therefore also be explained if α-SMA myofibroblasts have decreased already, while collagen has not been cleared yet. Since matrix metalloproteinases (MMPs) are normally mostly produced by macrophages (23,24), and macrophages were also decreased by rapamycin treatment in our study, it is possible that the deposited collagen was not cleared due to a lack of MMPs. However, an increased deposition of collagen by other cell types than myofibroblasts cannot be excluded based on our findings.

Rapamycin has previously been shown to directly induce autophagy in a variety of cell types in the kidney (25). Autophagy is known as a cell survival process during starvation. However, whether this process is beneficial in the IRI model is currently under debate (26–28). We used LC-3, a component of autophagosomes, as a marker for the induction of autophagy. We indeed observed a dramatic increase in autophagy in rats treated locally with rapamycin compared to systemic delivery. The increase in autophagy, regardless of its role in AKI, gives additional evidence for strong local inhibition of mTOR signaling after subcapsular delivery of rapamycin, suggesting higher local levels of rapamycin than those which were achieved after systemic delivery.

## Conclusion

The main goal of this study was to investigate whether local delivery of rapamycin using subcapsular injection of mMSP would be superior compared to systemic delivery. Based on our results we conclude that subcapsular implantation of mMSP itself does not induce adverse effects. Furthermore, rapamycin was effectively released from the mMSP and increased drug effects were seen after local delivery compared to systemic delivery from a subcutaneous mMSP depot. Thus injection of drug-containing monospheres underneath the renal capsule is a safe and useful method to increase drug effectivity.

## Electronic supplementary material

Figure 1S Effects of low-dose rapamycin after subcapsular delivery of rapamycin or placebo. Placebo MSP and rapamycin containing MSP were injected subcapsularly in a model of IRI and explanted at day 7. Average area was determined by morphometry. ED-1 staining was performed to assess renal interstitial macrophage infiltration (A). α-SMA staining was performed to detect renal interstitial myofibroblasts (B). Collagen I (C) staining was performed in order to assess renal interstitial extracellular matrix deposition. LC3 staining was performed in order to detect autophagy induction as a marker for rapamycin release (D). (GIF 34 kb)

High resolution image (TIFF 355 kb)

Figure 2S Renal staining for macrophages, myofibroblasts, collagen I, collagen III or LC-3 after subcutaneous and subcapsular delivery of rapamycin or placebo. Placebo MSP and rapamycin containing MSP were injected subcutaneously and subcapsularly in a model of IRI and explanted at day 14. Average area was determined by morphometry. ED-1 staining was performed to assess renal interstitial macrophage infiltration (A). α-SMA staining was performed to detect renal interstitial myofibroblasts (B). Collagen I (C), III (D) stainings were performed in order to assess renal interstitial extracellular matrix deposition. LC3 staining was performed in order to detect autophagy induction as a marker for rapamycin release (E). (GIF 91 kb)

High resolution image (TIFF 2784 kb)

## ABBREVIATIONS

AKI: Acute kidney injury
GFR: Glomerular filtration rate
HPF: High power field
IRI: Ischemia/reperfusion injury
MMPs: Matrix metalloproteinases
mMSP: Monodisperse microspheres
MSP: Microspheres
PC: Placebo subcapsular
PSc: Placebo subcutaneous
RCH: Rapamycin subcapsular high dose
RCL: Rapamycin subcapsular low dose
RSc: Rapamycin subcutaneous high dose
α-SMA: α-smooth muscle actin
