# Supplementary figures and images for: Microsphere-Based Rapamycin Delivery, Systemic Versus Local Administration in a Rat Model of Renal Ischemia/Reperfusion Injury
Source: Pharm Res. 2015 May 9;32(10):3238–47. doi: 10.1007/s11095-015-1700-8 (PMC4577552; doi:10.1007/s11095-015-1700-8)

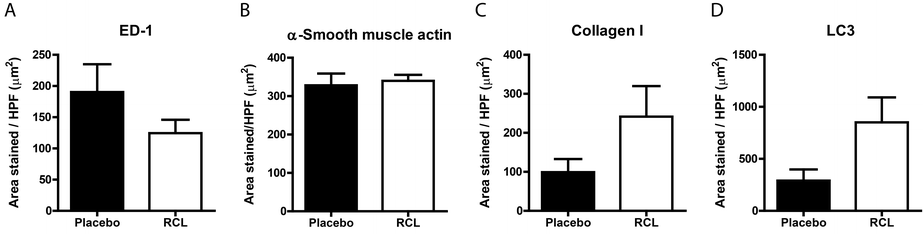

Supplement: Supplementary file 1 — Effects of low-dose rapamycin after subcapsular delivery of rapamycin or placebo. Placebo MSP and rapamycin containing MSP were injected subcapsularly in a model of IRI and explanted at day 7. Average area was determined by morphometry. ED-1 staining was performed to assess renal interstitial macrophage infiltration (A). α-SMA staining was performed to detect renal interstitial myofibroblasts (B). Collagen I (C) staining was performed in order to assess renal interstitial extracellular matrix deposition. LC3 staining was performed in order to detect autophagy induction as a marker for rapamycin release (D). (GIF 34 kb) [file 11095_2015_1700_Fig7_ESM.gif]

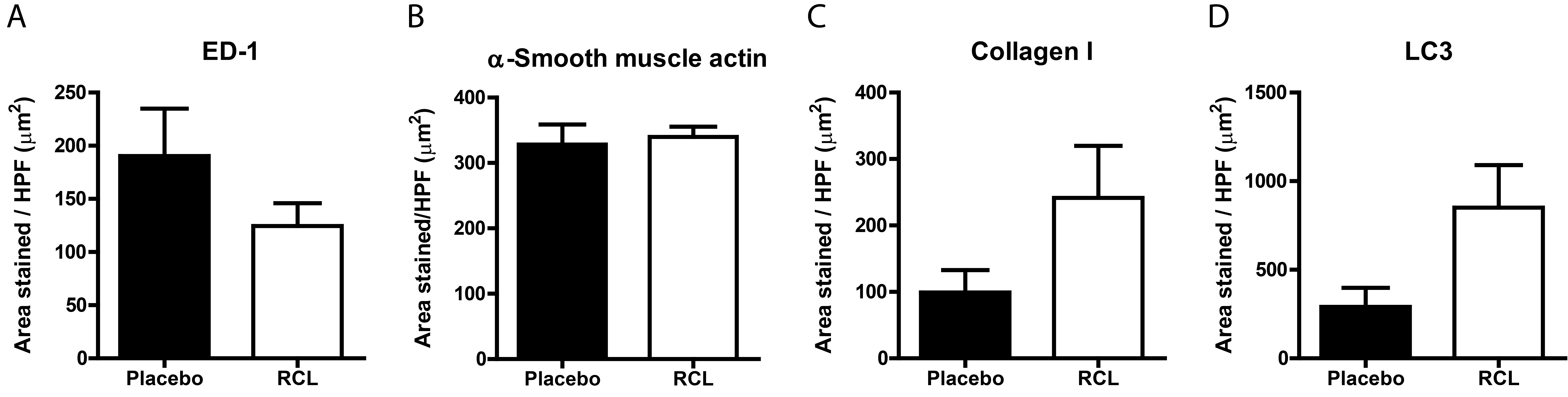

Supplement: Supplementary file 2 — High resolution image (TIFF 355 kb) [file 11095_2015_1700_MOESM1_ESM.tif]

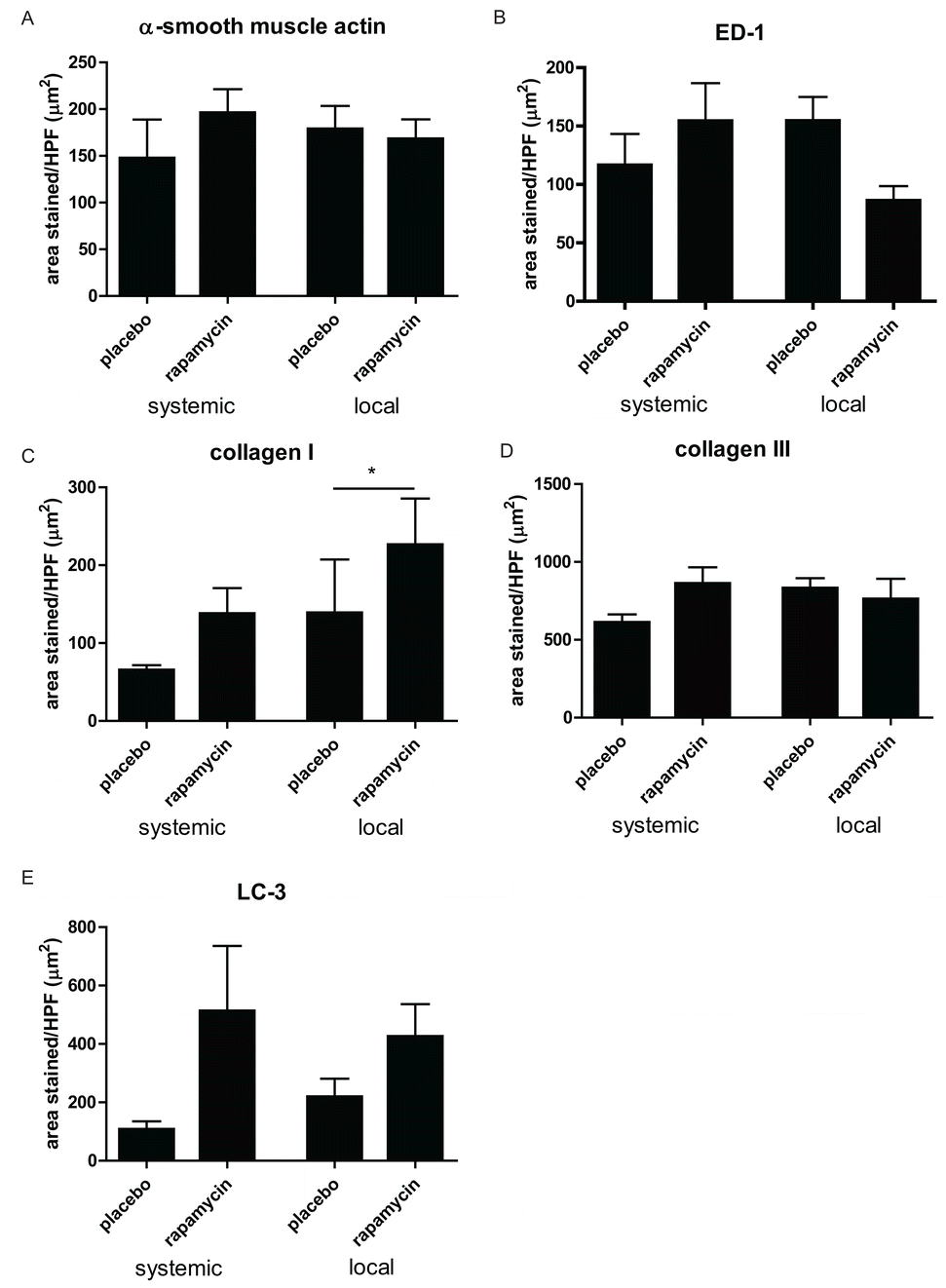

Supplement: Supplementary file 3 — Renal staining for macrophages, myofibroblasts, collagen I, collagen III or LC-3 after subcutaneous and subcapsular delivery of rapamycin or placebo. Placebo MSP and rapamycin containing MSP were injected subcutaneously and subcapsularly in a model of IRI and explanted at day 14. Average area was determined by morphometry. ED-1 staining was performed to assess renal interstitial macrophage infiltration (A). α-SMA staining was performed to detect renal interstitial myofibroblasts (B). Collagen I (C), III (D) stainings were performed in order to assess renal interstitial extracellular matrix deposition. LC3 staining was performed in order to detect autophagy induction as a marker for rapamycin release (E). (GIF 91 kb) [file 11095_2015_1700_Fig8_ESM.gif]
